# Platelets: New Bricks in the Building of Neutrophil Extracellular Traps

**DOI:** 10.3389/fimmu.2016.00271

**Published:** 2016-07-06

**Authors:** Agostina Carestia, Tomas Kaufman, Mirta Schattner

**Affiliations:** ^1^Laboratory of Experimental Thrombosis, Institute of Experimental Medicine-CONICET, National Academy of Medicine, Buenos Aires, Argentina

**Keywords:** platelets, neutrophils, neutrophil extracellular traps, inflammation, platelet–neutrophil interaction

## Abstract

In addition to being key elements in hemostasis and thrombosis, platelets have an important role in the inflammatory and innate immune response. This activity is associated with their capability to recognize pathogens through the expression of toll-like receptors, the secretion of various cytokines, chemokines, and growth factors stored within their granules, and the expression of cell adhesion molecules that allows interaction with other immune cells, mainly neutrophils and monocytes. As part of the first line of defense, neutrophils control invading pathogens by phagocytosis, the release of antimicrobial proteins during degranulation, or through the formation of web-like structures named neutrophil extracellular traps (NETs). NETs are formed by chromatin, proteases, and antimicrobial proteins, and their main function is to trap and kill bacteria, virus, and fungi, avoiding their dissemination. Besides microorganisms, NET formation is also triggered by proinflammatory molecules and platelets. The uncontrolled formation of NETs might exert tissue damage and has been involved in a pathogenic mechanism of autoimmune and prothrombotic clinical conditions. In this review, we discuss the role of platelets in NET generation highlighting the mediators, stimuli, and molecular mechanisms involved in this phenomenon, both in human and murine models.

## Introduction

Platelets are known to be central mediators of hemostasis and thrombosis; however, this role and the spatiotemporal relationship of the events that occur after tissue injury also link them directly to the host inflammatory and immune responses. The contribution of platelets to processes beyond hemostasis and thrombosis is associated with several properties, including the ability to store within intracellular granules – a broad array of biologically active molecules, which can be released into the circulation or translocated to the platelet surface during activation; and the recognition of pathogens through toll-like receptors (TLRs), and by the interaction of platelets directly with pathogens and other vascular cells, mainly endothelial cells and leukocytes (1).

The release of DNA extracellular traps is a newly discovered defense mechanism of neutrophils in their fight against pathogen invasion. Neutrophil extracellular traps (NETs) occur as a result of a special cell death program named NETosis that involves the release of DNA webs decorated with all five types of histones and with neutrophil-derived granular proteins with antimicrobial activity, such as elastase, myeloperoxidase (MPO), and the bactericidal/permeability-increasing protein, among others (2). Even though these traps are important as anti-infection agents in the innate immune response, they can be a double-edged sword since, in excess, they are cytotoxic and induce tissue damage (3).

Increasing *in vitro* and *in vivo* evidence indicates that the ability of platelets to operate as innate immune cells is critical to the process of NET formation. In this review, we aim to discuss the role of platelets in the formation of NETs, identifying the stimuli, mediators, and molecular mechanisms involved as well as their physiopathologic relevance, both in mice and humans.

## Platelets

Platelets are anucleated cells derived from megakaryocytes, which play a crucial role in hemostasis and thrombosis. Under normal conditions, circulating platelets do not adhere to the wall of blood vessels, leukocytes, or between them due to the anti-thrombotic properties of vascular endothelium. However, during vascular injury or after endothelium activation under inflammatory conditions, platelets adhere to subendothelial molecules such as collagen or von Willebrand factor (vWF), triggering initial platelet activation characterized by the release of soluble mediators stored in their granules, such as adenosine diphosphate (ADP) and thromboxane A_2_ (TXA_2_). These molecules act as platelet activators amplifying cell activation in an autocrine manner and allowing the activation of other circulating platelets, favoring the formation of platelet–platelet aggregates. The interaction between platelets is called aggregation and occurs due to the principal integrin of these cells, α_IIb_β_3_. Upon platelet activation, this integrin changes from a closed to an open conformation, enabling fibrinogen binding, which acts as a bridge between platelets. The amplification of platelet stimulation leads to procoagulant activity, thrombin generation, and formation of a thrombus that will repair the damaged vessel (4).

## Neutrophil Extracellular Traps

The main function of NETs is to eliminate pathogens. Once attached to these webs, histones and microbicide proteins degrade virulence factors and kill pathogens. The NET is not only a trap, which prevents the propagation of pathogens from the initial site of infection, but also, by being attached to injurious stimuli, concentrates microbicide proteins to the site of infection (2). While NET formation was initially associated with pathogens such as bacteria, fungi, viruses, and parasites (5–9), it is now known that cytokines, chemokines, platelet agonists, and antibodies (10–14) are also capable of triggering this phenomenon. Depending on the location of the neutrophils when stimulated (extravasated versus vascular), these NETs can be either spread throughout the interstitium of specific organs or released into the lumen of blood vessels, where they may attach to the vessel walls of narrow capillaries.

NETosis is initiated by the activation of peptidylarginine deiminase 4 (PAD4), which induces citrulination of histones 3 and 4 dismantling the nucleosome (15, 16). In addition, the activation of protein kinase C and nuclear factor kappa B (NFκB) pathways (12) and RAF/MEK/ERK signaling (17) leads to phosphorylation of several kinases resulting in assembly of the functional nicotinamide adenine dinucleotide phosphate (NADPH) oxidase complex for reactive oxygen species (ROS) generation (18). ROS trigger the dissociation of neutrophil elastase from a membrane-associated complex (azurosome) into the cytosol and activate its proteolytic activity in an MPO-dependent manner. Once in the cytoplasm, elastase binds actin and is translocated to the nucleus where it promotes chromatin decondensation by cleavage of histones. After disintegration of the nuclear envelope and rupture of the cytoplasmic membrane, NETs are released (19, 20).

In addition to ROS generation, autophagy is also required for NET generation (21). Furthermore, recent evidence has also shown that activation of SK3 potassium channels, mediated by calcium influx, may lead to an alternative NADPH oxidase-independent mechanism of NETosis (22).

The above description is of what is called “suicidal” NETosis (6). However, it has been reported that a fraction of neutrophils can release NETs without dying, leaving behind cytoplasts that continue to ingest microbes. This highly efficient process is called “vital” NETosis (23, 24).

NETs form an important arm of antimicrobial innate immunity, which is exemplified by the fact that defects in NET generation, or experimental NET depletion, increase susceptibility to various kinds of infections in mice and humans (25–28).

## Role of Platelets in Net Formation

Platelet involvement in DNA extracellular trap formation was first described in 2007, when Clark et al. showed in a mouse model of sepsis that lipopolysaccharide (LPS) binds to TLR4 present in the platelet membrane, allowing the binding of platelets to neutrophils and leading to rapid neutrophil activation and NET formation. These structures maintain their integrity under flow conditions and ensnare bacteria in the circulation (29). The finding that activation of TLR4 on platelets activates the release of NETs was a significant shift in understanding the inflammatory response to sepsis. The ability of platelets to promote NET formation was also observed in human cells, not only with Gram-negative bacterial components but also with Gram-positive bacteria (14).

Platelet-mediated NET formation is not restricted to activation by bacteria; it has been shown that platelets are also required for NETosis-mediated virus clearance. After systemic poxvirus challenge, mice exhibit thrombocytopenia and the recruitment of both neutrophils and platelets to the liver vasculature. Circulating platelets interact with, roll along, and adhere to the surface of adherent neutrophils, forming large aggregates that facilitate the release of NETs within the liver vasculature. Inhibition of this aggregation does not form NETs (30). The platelets’ participation in the formation of NETs and virus clearance could represent another mechanism for the thrombocytopenia that is frequently observed in virus-infected patients (31). Although the central role of platelets in NETosis was initially identified in infectious processes, it also happens during sterile inflammation conditions. In fact, activation of platelets, not only by pathogens but also by classical platelet agonists such as thrombin, arachidonic acid, collagen, or ADP, results in NET induction (11, 13, 14, 32). These findings, together with the fact that besides exerting antimicrobial activity, NETs can also trigger procoagulant, prothrombotic, and proinflammatory responses and explain why NETs are increasingly being recognized as novel mediators of several non-infectious clinical conditions, such as thrombosis, pre-eclampsia, and autoimmune diseases (3).

## Cell Adhesion Molecules Involved in Platelet-Mediated NETosis

Platelet–neutrophil interaction takes place mainly due to the binding of P-selectin to its receptor P-selectin glycoprotein ligand-1 (PSGL-1) on the leukocyte surface (33). Consistent with this observation, recent studies in mice have shown that NET formation during sepsis or acute lung injury (ALI) experimental models is also dependent on cell to cell interaction *via* PSGL-1 and P-selectin (34, 35). However, other studies, including those from our group, have indicated that, in humans, platelet-mediated NET formation is a P-selectin-independent mechanism since its blockade does not modify DNA release and the incubation of neutrophils with recombinant P-selectin was not able to induce NETosis (13, 14). Platelet TLR4 appears to be a major target involved in platelet-mediated NETosis. As mentioned before, platelet TLR4 is involved in inducing NETs in mice and humans (14, 29). However, it is not yet known whether the LPS bound to platelet TLRs interacts with the neutrophils’ TLR4 counter receptor, or if it is just necessary to trigger platelet activation and the release of mediators that, in turn, induce NETosis. Of note, the blockade of platelet TLR4 markedly impairs NETosis and this has been suggested as a new therapeutic approach for sepsis (29).

Cooperation between neutrophil β2 integrin (CD18) and platelet glycoprotein (GP) Ib has been considered as another major event involved in platelet–neutrophil adhesion (36, 37). Studies using murine cells of wild type and mice deficient in CD18, as well as a murine model of sepsis, demonstrated that β2 integrin is also involved in platelet-mediated NETosis. Furthermore, blocking Mac-1 (CD18/CD11b), but not LFA-1 (CD18/CD11a), in wild-type mice significantly reduced NET formation (38, 39). These data were corroborated in studies of mice and human cells where blocking CD18 markedly decreased the release of NETs mediated by activated platelets (14, 39). With regard to platelet GP Ib, its specific inhibition by gene deletion (40), or using blocking antibodies in human platelets (14), also affected the formation of NETs, demonstrating its participation in this process.

In a mouse model of ALI and using mice platelets and neutrophils, it has been shown that platelet-stimulated NETs require simultaneous activation of integrin-mediated outside-in and G-protein-coupled receptor (GPCR) signaling. Blocking one of these pathways reduced NET formation *in vivo*, diminishing the severity of ALI (39).

Platelet integrin α_IIb_β_3_ is the most abundant molecule in the platelet’s surface that mediates interaction between platelets, and platelets with other cells. In a mouse model of lung injury, blocking the platelet integrin inhibited the formation of NETs and tissue damage (11), whereas a study of human platelets showed that α_IIb_β_3_ seems not to participate in this process (13, 14).

Together, these observations suggest that the bridging between platelets and neutrophils in NET formation differs either between species or due to *in vivo* cofactors not involved in the *in vitro* models. In fact, while platelet-mediated NETosis in mice appears to be dependent on P-selectin, GPIb, TLR4, and integrin α_IIb_β_3_ expression on the platelet surface; human NET formation by platelets seems to only require GPIb and TLR4. In contrast, integrin β2 on the neutrophil’s surface appears to be a major protein involved in platelet-mediated NETosis, either in mice or humans.

## Soluble Mediators of Platelet-Mediated NETosis

Neutrophils and platelets activate each other. The crosstalk between these cells depends on cell-to-cell contact and secreted substances. Upon activation, platelets express or secrete various molecules capable of modulating the activation of neutrophils (41).

High mobility group box 1 (HMGB1) is a damage-associated molecular pattern molecule, which is released as a result of cell death and represents a crucial sign in the inflammatory response to tissue injury (42). Platelets contain HMGB1, which is released after activation (43). Recently, it has been shown that platelet-derived HMGB1 is a mediator of NET formation. Of interest, HMGB1, either soluble or presented from activated platelets, induces autophagy in neutrophils promoting NETosis and inhibiting apoptosis (13), supporting the notion that autophagy is a critical signal for the NETosis process. Of note, NETs induction by HMGB1 is independent of TLR4; thus, the axis HMGB1–receptor for advanced glycation endproducts (RAGE) represents a potential target to control NET formation in sterile inflammatory conditions.

Platelet-derived *chemokines* such as RANTES (CCL5) and platelet factor 4 (CXCL4, PF4) are well-known *molecules* responsible for neutrophil recruitment to sites of inflammation/infection (44). In a mouse model of lung injury, it has been shown that blocking these cytokines or their heterodimerization inhibited NETosis (39). We have confirmed the participation of PF4 in NETosis using human cells, while inhibition of PF4 abrogated the release of DNA, recombinant PF4 promoted the formation of these DNA traps (14). These observations suggest that, besides chemotaxis, RANTES and PF4 are involved in the inflammatory response, not only recruiting leukocytes to the site of injury but also being strong NETs inducers.

Venous thromboembolism is a GP stored in α granules of megakaryocytes and platelet and Weibel Palade bodies of endothelial cells. After vessel injury, vWF mediates platelet adhesion to the exposed subendothelium (45). Several studies have suggested that vWF is a critical component of NETs. In a murine model of deep vein thrombosis (DVT), it was observed that citrullinated histone H3 (citH3) colocalized with this factor, suggesting that this molecule and NETs form a network that contributes to thrombus growth and stabilization (46). Furthermore, the ultralarge vWF fibers adhered to the endothelium that are observed during inflammation, bind and immobilize the DNA released by neutrophils acting as a linker between leukocyte adhesion to the endothelium and supporting leukocyte extravasation and inflammation (47). In addition, we found that incubation of human platelets with an antibody against vWF suppressed NET formation induced by activated platelets, indicating that not only vWF is a NET inducer but also revealing a functional role for this molecule is released by platelets (14). Together, these observations indicate that, besides its function as an adhesive molecule, vWF secreted either by platelets or endothelial cells has a central role in the inflammatory and thrombotic features of NETs.

**Figure 1:**
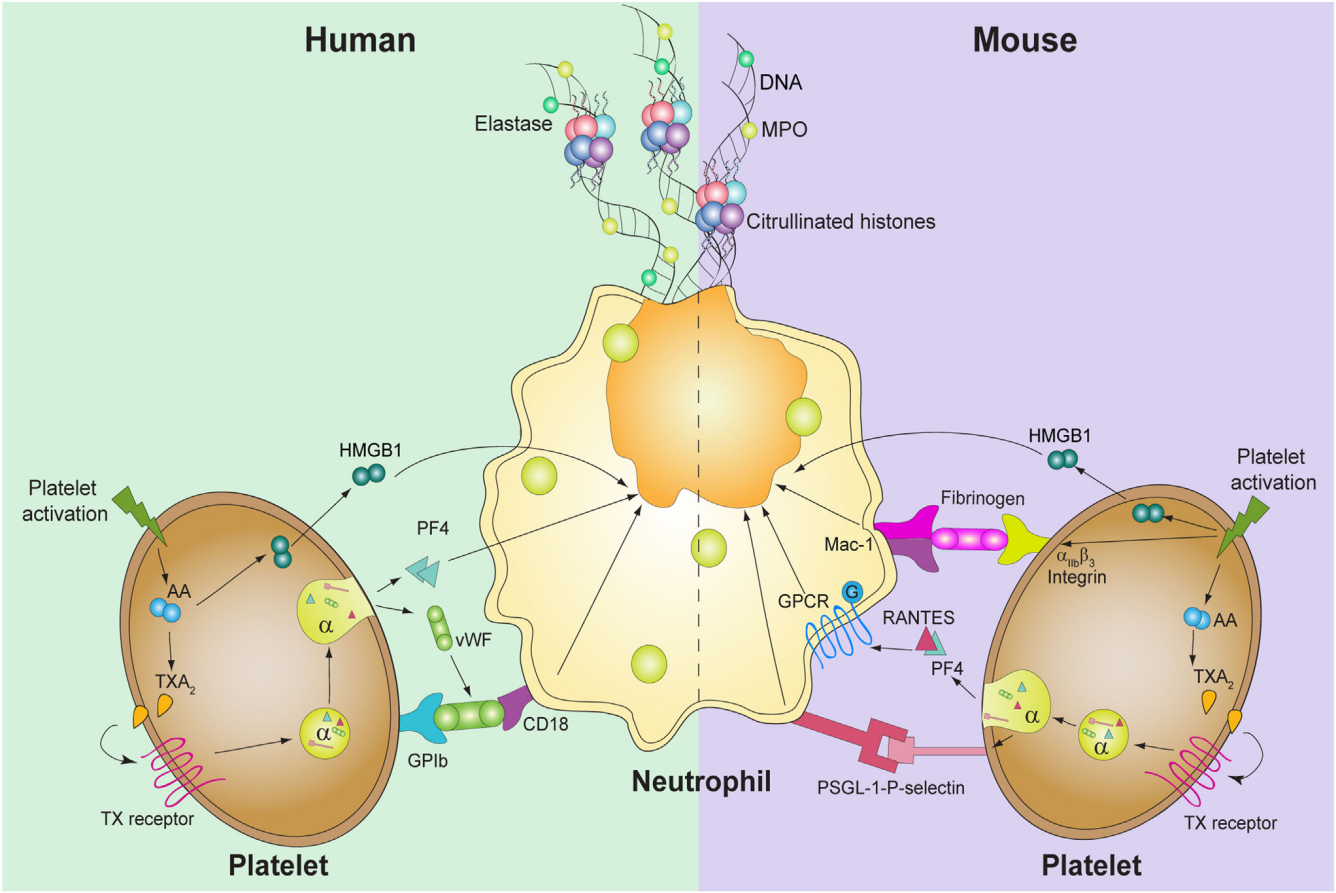
**Known molecules involved in platelet-mediated NET formation**. In humans (left), platelet activation induces thromboxane A_2_ (TXA_2_) formation, which triggers the release of high mobility group box 1 (HMGB1), von Willebrand factor (vWF), and platelet factor 4 (PF4). vWF binds to its platelet receptor, glycoprotein (GP)Ib, which binds to CD18 in neutrophils. PF4 and HMGB1 act directly upon neutrophils and induce DNA release. In mice, not only TXA_2_ generation and HMGB1 are involved but also PF4 and RANTES, through GPCR, induce platelet-mediated NET formation. Also, the interaction between P-selectin-PSGL-1 and α_IIb_β_3_-Mac-1 is required for the release of NETs.

Thromboxane A2 is a potent platelet agonist, and its role in the formation of platelet-mediated NETs has been recently studied. In this context, it was reported that pharmacological inhibition of TXA_2_-mediated platelet activation by acetylsalicylic acid (ASA) suppressed the formation of platelet-mediated NET generation (11, 14). Furthermore, pretreatment of neutrophils with a selective thromboxane receptor antagonist (SQ29548) prior to the addition of thrombin receptor activating peptide (TRAP)-activated platelets reduced the production of NETs (11). The *in vivo* relevance of TXA_2_ in platelet-mediated NET formation was first pointed out by Caudrillier et al. who showed in experimental transfusion-related acute lung injury (TRALI) that NETs are detectable in the lung microcirculation and they were prevented by inhibition of platelet aggregation using ASA (11). In addition, dipetalodipin and triplatin are triatomine salivary proteins that exhibit high affinity binding to prostanoids, such as TXA_2_, inhibited platelet-mediated NET formation (32). Given that neutrophils do not express the TXA_2_ receptor, the effect of TXA_2_ is intriguing. However, as TXA_2_ is a strong autocrine and paracrine activator, we have shown that this molecule, once released, acts on platelets triggering the release of platelet granules content, which in turn promote the formation of NETs (14) (Figure 1).

## Signaling Involved in Platelet-Mediated Net Formation

**Figure 2:**
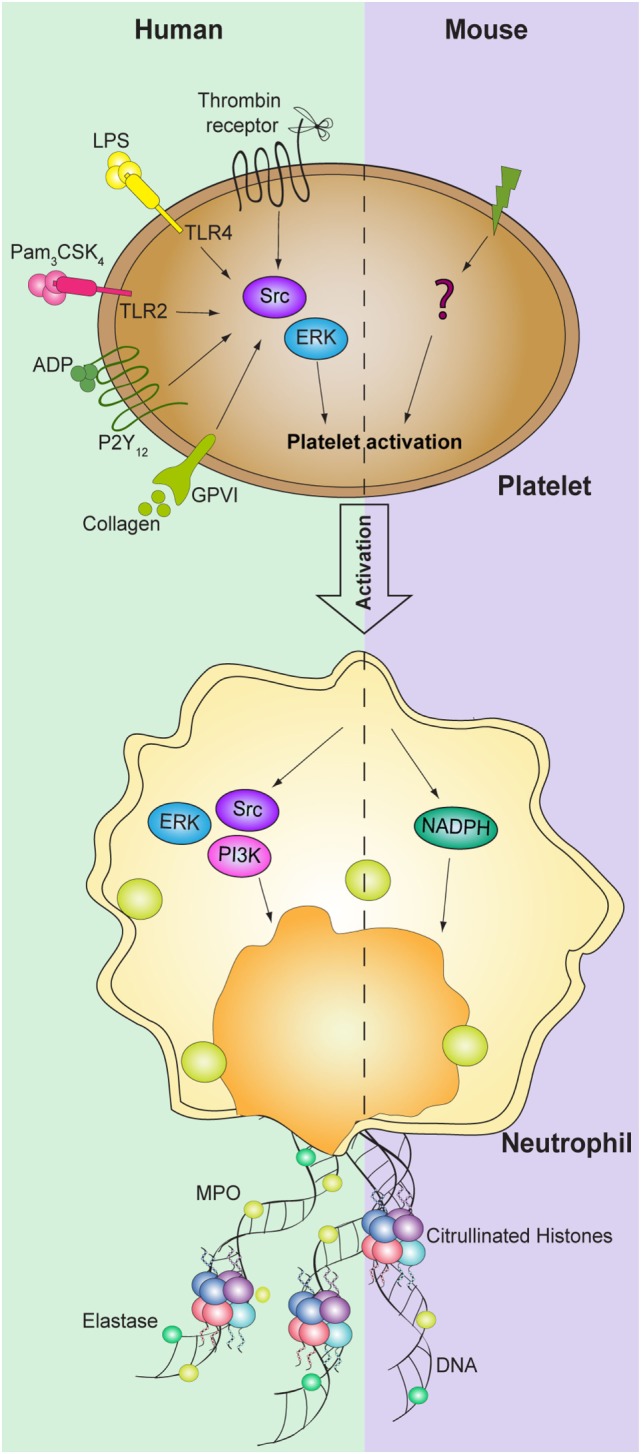
**Signaling pathways involved in platelet-mediated NET formation**. NETosis mediated by platelets requires activation of both human platelet and neutrophils ERK and Src kinases. In addition, PI3K signaling pathway in neutrophils is also required for NET formation. While NADPH oxidase in neutrophils is necessary to induce NET formation in mice, the signaling pathways in platelets are still not identified.

Although the molecular basis governing the formation of NETs are not yet fully elucidated, the activation of the RAF/MEK/ERK, the generation of ROS by the enzyme NADPH oxidase, and activation of PAD4 seems to be of great importance in the NETosis induced by phorbol-12-myristate 13-acetate (PMA) (15–18). Phosphorylation of ERK both in platelets and neutrophils is also necessary for the formation of NETs mediated by activated platelets (11, 14, 48). Blocking ROS production, in both cell types, did not influence the release of DNA traps in human cells (14); however, in a murine model, NETosis was significantly decreased using diphenyleneiodonium (DPI) both *in vitro* and *in vivo* (39). Nevertheless, while platelet activation of Src is required for induction of platelet-mediated NETosis, in neutrophils both Src and PI3K activation are triggered during NET formation mediated by platelets (14, 48) (Figure 2). Further studies are required to elucidate the signaling pathways involved in platelet-mediated NETosis.

## Platelets and NETs in Disease

### Infectious Inflammation

#### Sepsis

It is increasingly recognized that platelets play an important role in the host defense to pathogens. Several mechanisms appear to be responsible for this response, including the ability of platelets to directly recognize, sequester, and kill pathogens, to recruit leukocytes to the site of infection and to activate neutrophils, enhancing their ability to phagocytose and kill pathogens (49). In addition, a seminal study by Clark et al. (29) demonstrated that platelets induce neutrophil unique effector responses, such as the generation of NETs. In response to endotoxemia, neutrophils adhere to endothelium mainly in the sinusoids of the lungs and liver and then LPS-activated platelets anchor to the surface of neutrophils, promoting NETosis. Because these interactions were observed during severe sepsis, it was suggested that platelets, through the expression of TLR4, act as a barometer for systemic infection and, under high levels of LPS, the interplay between platelets and neutrophils constitutes an efficient mechanism in the fight against pathogens. Remarkably, the formation of NETs, if uncontrolled, is also responsible for tissue damage and organ dysfunction. The biological relevance of platelets in NET formation during sepsis was underscored by the observation that plasma from severely septic patients also triggered the formation of neutrophil DNA traps in the presence of platelets (29). In addition, in a *Streptococcus mutans* endocarditis rat model, it has been demonstrated that infective endocarditis-inducing pathogens activate platelets through specific IgG, which contributes to bacteria–platelet aggregate formation on the damaged valve; the bacteria also activate the infiltrated neutrophils and produce ROS, which triggers chromatin decondensation. The resulting NETs entrap the bacteria–platelets aggregates to promote vegetation formation (48). These observations lead the authors to suggest that a similar process could take place in human endocarditis, because NETs have been identified in human specimens.

Interestingly, it was recently proposed that the link between coagulation and the innate immune response might constitute an innate effector mechanism, named immunothrombosis, which mediates the recognition of pathogens and damaged cells and inhibits pathogen dissemination and survival. Immunothrombosis is therefore considered to be a physiological process crucial for intravascular immunity, whereas its dysregulation may be one of the underlying events that trigger thrombotic disorders, such as VTE and disseminated intravascular coagulation. Neutrophils are major players in the development of immunothrombosis through the release of NETs. Moreover, NETs may also provide a scaffold to activate platelets and stimulate thrombus formation (50). Several studies have indicated the relationship between infection and thrombosis; however, the pathogenic mechanism was not clear. The discovery of NETs and the bidirectional interaction with platelets represents a link between these phenomena.

### Sterile Inflammation

#### Acute Lung Injury

In a murine model of TRALI, NETs were found in the microcirculation of the lungs and components of these networks in plasma (11). When platelet function was inhibited, DNA content significantly decreased indicating that platelets are necessary for the generation of NETs. Similar results were demonstrated in a mouse model of ventilation-induced lung injury (39). In this study, platelet depletion resulted in a decreased neutrophil accumulation in alveolar compartments and release of these DNA traps. Furthermore, a similar result was obtained by specifically inhibiting the heterodimerization of RANTES and PF4, reaffirming the role of platelets in this mechanism, and increasing the severity of the disease. However, Thomas et al. reported that platelet depletion with neuraminidase in a murine model of TRALI did not prevent NET formation in the lungs (51). This observation was only qualitative, thus, it is conceivable that platelets potentiate the release of NETs induced by other agonists. Taking into account these discrepancies, more studies are needed to understand the relevance of platelets in the formation of NETs in TRALI and whether therapeutic inhibition of platelet–neutrophil aggregates and/or NET formation provide a useful therapeutic strategy.

#### Thrombosis

##### Venous Thrombosis

Traditionally, the pathogenic mechanism of venous thromboembolism (VTE) that includes DVT or pulmonary embolism was considered the result of reduction in blood flow, injury to the vascular endothelium and a hypercoagulable state (Virchow’s Triad) (52). The discovery of NETs offered a very exciting explanation for the cellular events that trigger VTE. Initial studies showed that DNA traps promote platelet adhesion, activation, and aggregation and exert procoagulant activity, thus providing a scaffold for thrombus formation and growth (53). These effects are mainly caused by histones present in the NETs (54–56). Moreover, histones induce the release of platelet mediators that, in turn, induce NETosis, thus creating a positive feedback mechanism that favors thrombus formation. NETs also recruit red blood cells and promote vWF, fibrinogen, and fibrin deposition inducing a red thrombus, such as that found in veins. Remarkably, clot formation in the presence of activated neutrophils can be prevented only by simultaneous treatment with tissue plasminogen activator and deoxyribonuclease (DNAse), indicating that NETs may provide a scaffold independent from fibrin (53).

Using a mouse model of flow restriction-induced DVT, von Bruhl et al. and Brill et al. demonstrated that flow restriction triggers the rapid accumulation of leukocytes, mainly neutrophils (85%) and monocytes (15%), that initiate local fibrin formation through the expression of tissue factor. In addition, neutrophils form NETs that bind factor XII and initiate coagulation. Platelets were critical to foster neutrophil recruitment and NET formation through the GPIb-β_2_ integrin axis and supported fibrin formation by enhancing neutrophil-dependent coagulation. While DNAse I and enoxaparin protected mice from DVT, infusion of an unfractionated mixture of calf thymus histones increased plasma vWF and promoted DVT early after stenosis application, indicating the relevant role of NETs in DVT (40, 46). In fact, these experimental models provided the first *in vivo* evidence that NETs can act as a platform for thrombogenesis in large veins in response to perturbations of flow and also that these DNA traps are a unique link between inflammation and DVT. Furthermore, these studies highlighted that platelets, commonly recognized as central mediators of arterial thrombosis, also contribute to inflammation and the propagation of DVT in the absence of endothelial disruption, mainly fostering NET formation.

The critical role of NETs in developing DVT was also demonstrated by Martinod et al., who showed that PAD4-deficient mice (PAD4^−^/^−^) were protected from producing venous thrombi (57). However, in contrast to these observations, El-Sayed et al. found no differences in the stasis thrombus size either in WT animals that were treated with DNAse I or in PAD4^−/−^ mice compared with controls (58). The differences between these studies are still not clear and require further investigation. Furthermore, unexpectedly, it was recently reported that neutrophils from elastase-deficient mice only showed a mild reduction in non-infectious stimuli-mediated NET formation *in vitro* and accordingly, elastase deficiency had no significant effect on thrombosis in the inferior vena cava stenosis model (59). These data revealed not only that neutrophil elastase is not essential for NETosis but also that even partial NET release seems sufficient to attract platelets, red blood cells, promote coagulation, and drive venous thrombus formation. Since all these findings were obtained in mice, the question arises whether similar molecular mechanisms are effective in humans.

The first clinical study of 150 patients showed that increased levels of circulating nucleosomes and neutrophil activation, as evidenced by the presence of elastase–antitripsin complexes, were associated with a threefold risk of DVT (60). In another study, immunohistochemical analysis of thrombi obtained from patients at different stages of VTE demonstrated the presence of NETs during the organizing stage of thrombus maturation. In contrast, NETosis was absent in the mature regions of organized thrombi. CitH3-positive cells surrounded vWF-positive platelet islands in human DVT samples, and diffuse vWF-positive staining was associated with diffuse citH3 patterns in the organizing regions of thrombi (61). These findings support the notion that NETs, together with vWF, could enhance thrombus formation/stability in DVT.

##### Arterial Thrombosis

Increased levels of nucleosomes and cell-free DNA have frequently been shown to be associated with infarcted tissue in patients with ischemic stroke and myocardial infarction (MI) (62). However, because these biomarkers are not specific to tissue injury, it was not clear from these studies whether the DNA material was released from necrotic tissues or from NETs. Furthermore, the relationship was not established between the nucleosome of cell-free DNA and thrombi. The presence of citH3 neutrophils in heart sections and increased plasma nucleosomes levels in mice subjected to myocardial reperfusion injury was recently demonstrated. Also of note, monotherapy with DNAse I significantly decreased the number of infiltrating neutrophils and plasma nucleosomes levels as well as reducing the infarcted area and improving cardiac function, indicating not only that NETs are components of the inflammatory milieu of the infarcted myocardium but also their direct involvement in cardiac injury (63). In a prospective, observational, cross-sectional cohort of 282 individuals with suspected coronary artery disease, Borissoff et al. found that markers of NETs, such as MPO–DNA complexes, predicted the number of atherosclerotic coronary vessels, the presence of a hypercoagulable state, and the occurrence of major adverse cardiac events (64). Moreover, they also identified high nucleosome levels as an independent risk factor of severe coronary stenosis. Of note, this study only found a weak correlation of platelet activation markers (measured by PF4 plasmatic levels) and citH4 levels. However, in another study, Maugeri et al. demonstrated that in patients with acute MI, coronary artery thrombi obtained during percutaneous coronary intervention were made up mainly of platelets and neutrophils and contained large amounts of DNA strands that were decorated with citrullinated histones and granule proteins such as MPO and pentraxin; platelets were identified close to DNA webs (13). In support of these observations, citH3 was detected in the cerebral microthromboses of ischemic stroke patients. Interestingly, the patients also had an unexpectedly higher prevalence of cancer; half of the cancers were diagnosed post-mortem (65). Furthermore, cancer patients had increased plasmatic markers of NETosis that correlated positively with thrombin–antithrombin levels and soluble P-selectin. These data not only further support the link between NETosis and thrombosis but also highlight the relevance of NET formation as biomarkers detecting cancer in arterial microthrombosis, or vice versa.

The role of NETs and platelets was also confirmed in patients with MI in selective samplings of thrombotic material and surrounding blood from the infarct-related coronary artery during primary percutaneous revascularization. The study showed that interaction of thrombin-activated platelets with neutrophils at the site of plaque rupture during an acute MI results in local NET formation and delivery of functional tissue factor (66).

## Conclusion

The presented evidence indicates that platelets, when stimulated by bacteria, viruses, or traditional agonists, are capable of binding to neutrophils, activating them to release NETs. This interaction can be beneficial to protect the host against pathogens but, if uncontrolled, can cause tissue and organ damage. Of note, while experiments performed *in vitro* are compelling in their collective confirmation that platelet–neutrophil interactions can be induced to generate NETs, the relevance of this interaction *in vivo* still contains elements of ambiguity and leads to numerous unresolved questions often involving pathway differences, both between species and cell types.

The interaction of platelets and neutrophils in NETosis induction appears to be a relevant pathogenic mechanism for both arterial and venous thrombosis; prevention of platelet- and neutrophil-mediated thrombogenesis is now being considered as a potential promising new target for anti-thrombotic therapy.

Although we have witnessed great advances during the last decade in the comprehension of NETosis and the involvement of platelets in this process, several questions still remain to be answered: are circulating nucleosomes and activated neutrophils causative of thrombus formation or are they rather a consequence of its development? Is there any degree of selectivity regarding the decondensation and release of DNA during NETosis mediated by platelets in sterile or infectious conditions? Is there any relationship between platelets and NETs formation in atherosclerotic plaque progression? Is NETosis mediated by platelets required for hemostasis?

It is clear that more experimental and clinical studies are required to further understand the physiopathologic relevance of this newly discovered interaction between platelets and neutrophils.

## Author Contributions

All authors listed have made substantial, direct, and intellectual contribution to the work and approved it for publication.

## Conflict of Interest Statement

The authors declare that the research was conducted in the absence of any commercial or financial relationships that could be construed as a potential conflict of interest.

## Abbreviations

ADP, adenosine diphosphate; ALI, acute lung injury; ASA, acetylsalicylic acid; DNAse, deoxyribonuclease; DVT, deep vein thrombosis; GP, glycoprotein; GPCR, G-protein-coupled receptor; HMGB1, high mobility group box 1; LPS, lipopolysaccharide; MI, myocardial infarction; MPO, myeloperoxidase; NADPH, nicotinamide adenine dinucleotide phosphate; NETs, neutrophil extracellular traps; PAD4, peptidylarginine deiminase 4; PF4, platelet factor 4; PMA, phorbol-12-myristate 13-acetate; PSGL-1, P-selectin glycoprotein ligand-1; ROS, reactive oxygen species; TLR, toll-like receptor; TRALI, transfusion-related acute lung injury; TXA_2_, thromboxane A2; VTE, venous thromboembolism; vWF, von Willebrand factor.
